# Sendai Framework’s Global Targets A and B: Opinions from the Global Platform for Disaster Risk Reduction’s Ignite Stage 2019

**DOI:** 10.1007/s13753-022-00432-3

**Published:** 2022-09-05

**Authors:** Nibedita S. Ray-Bennett, Krishna Clarke, Daniel Mendez

**Affiliations:** 1 School of Business, University of Leicester, Leicester, LE1 7RH UK; 2 Avoidable Deaths Network, University of Leicester, Leicester, LE1 7RH UK; 3 Office of the President Saint Michael, Parish of Saint Michael, Caribbean Development Bank, Bridgetown, 11000 Barbados

**Keywords:** Disaster deaths, Disaster risk reduction, Global Platform for Disaster Risk Reduction, Health-EDRM, Health system, Sendai Framework’s targets A and B

## Abstract

The Sendai Framework for Disaster Risk Reduction 2015−2030 set seven global targets of which the first two targets are to reduce disaster deaths (target A) and diminish the number of affected people globally (target B) by 2030. To realize these targets, the United Nations General Assembly’s Expert Working Group provided indicators to measure progress as well as terminologies for these targets in 2017. Research around these targets is nascent. This article contributes to the understanding of the targets by exploring: (1) what are the conditions that may hinder achieving targets, as well as those that may accelerate their achievement at the national and local levels; and (2) which types of organizations should lead a country’s effort to reduce disaster deaths? These questions were answered by opinion survey research carried out at the Sixth Session of the Global Platform for Disaster Risk Reduction. The participants identified disaster risk reduction efforts, early warning systems, awareness, finance and investment (among others) as the important facilitating factors to achieve targets A and B. Minimal investment in human security, lack of response and coordination, uncertainty of climate change, poor information, lack of campaigns and low budget allocation (among others) are considered as the important hindering factors for these targets by the participants. The findings also suggest that the facilitating and hindering variables of targets A and B are interconnected with global target E (disaster risk governance and capacity building). The majority of the participants thought that it is the national government who should lead in a country’s effort to reduce disaster deaths. Based on these findings, a few recommendations have been made to improve policy and practice related to the indicators as well as to reimagine theories so that targets A and B can be realized in alignment with target E at the national and local levels by 2030.

## Introduction

In 2015, the United Nations’ Sendai Framework for Disaster Risk Reduction 2015−2030 (hereafter, Sendai Framework) set seven global targets, of which the first two targets are: “(A) Substantially reduce global disaster mortality by 2030” and (B) “Substantially reduce the number of affected people globally by 2030” (UNISDR 2015, p. 9). After the launch of the Sendai Framework, the United Nations General Assembly established the Expert Working Group in 2017 to develop the indicators and terminologies essential for achieving these targets (UN General Assembly 2016; UNISDR 2017). Research around these targets is nascent. This article aims to contribute to the understanding of these targets and indicators further by exploring: (1) what are the conditions that may hinder achieving these targets, and those that may facilitate their achievement at the national and local levels; and (2) which types of organizations should lead a country’s efforts to reduce disaster deaths? To answer these questions, a semistructured questionnaire and key informant interviews were conducted with the participants of the Ignite Stage Session at the United Nations Office for Disaster Risk Reduction’s (UNDRR) Sixth Session of the Global Platform for Disaster Risk Reduction (hereafter, Global Platform) in 2019, in Geneva, Switzerland. Although the opinions of the participants are subjective, they provide a vantage point from which to rethink policy and practice that will be required to achieve targets A and B at the national and local levels by 2030.

The subsequent section provides an overview of the conditions and stakeholders that may facilitate and hinder targets A and B to contextualize the data. Following this, data collection and data analysis strategies are discussed, ending with a few implications for theory and practice in the concluding section.

## Overview of Conditions and Stakeholders for Sendai Framework Targets A and B

This section provides an overview of two enabling conditions based on the existing and emerging literature that can facilitate the achievement of Sendai targets A and B. This section also provides the stakeholders approach as envisaged by the Sendai Framework to reduce disaster deaths. In doing so, we tease out some of the hindrances of or limitations to target achievement.

After the launch of the Sendai Framework, the United Nations General Assembly’s Expert Working Group in 2017 promoted an enabling environment by providing “a set of possible indicators to measure global progress in the implementation of the Global Targets” (UN General Assembly 2016, p. 2), and made “recommendations for indicators and terminology” (UNISDR 2017, p. 3). See Table 1 for the indicators of targets A and B, and Table 2 for the terminologies: death, missing, and directly or indirectly affected. Targets and terminologies are important for operational clarity for national and international organizations. The targets to reduce disaster deaths and the number of people affected by disasters were missing in the Hyogo Framework for Action 2005−2015—the Sendai Framework’s predecessor (Ray-Bennett 2018a, 2018b).

**Table 1:** Global targets A and B of the Sendai Framework for Disaster Risk Reduction

Target A		Target B	
No.	Indicators	No.	Indicators
A-1	Number of deaths and missing persons attributed to disasters, per 100,000 population	B-1	Number of directly affected people attributed to disasters, per 100,000 population
A-2	Number of deaths attributed to disasters, per 100,000 population	B-2	Number of injured or ill people attributed to disasters, per 100,000 population
A-3	Number of missing persons attributed to disasters, per 100,000 population	B-3	Number of people whose damaged dwellings were attributed to disasters
		B-4	Number of people whose destroyed dwellings were attributed to disasters
		B-5	Number of people whose livelihoods were disrupted or destroyed, attributed to disasters

*Source* UNISDR (2017)

**Table 2:** Terminologies defined by the United Nations Office for Disaster Risk Reduction and the Expert Working Group in 2017

**Terminologies**	**Definitions**
Death	“[…] the number of people who died during the disaster, or directly after, as a direct result of the hazardous event.” (p. 8)
Missing	“Number of people whose whereabouts is unknown since the hazardous event. It includes people who are presumed dead, for whom there is no physical evidence such as a body, and for which an official/legal report has been filed with competent authorities.” (p. 8)
Directly affected^a^	“People who have suffered injury, illness or other health effects; who were evacuated, displaced, relocated; or have suffered direct damage to their livelihoods, economic, physical, social, cultural and environmental assets.” (p. 18)
Indirectly affected	“People who have suffered consequences, other than or in addition to direct effects, over time due to disruption or changes in economy, critical infrastructures, basic services, commerce, work or social, health and physiological consequences.” (p. 19)

*Source* UNISDR (2017).

^a^Recognising the difficulties of assessing the full range of all affected people (direct and indirect), the Expert Working Group recommended the use of an indicator that would estimate “directly affected” as more feasible than collecting data on indirectly affected (UNISDR 2017, pp. 19−20).

The term “directly affected,” uses widely available data, and according to the Expert Working Group, it can be used consistently across countries and over time to allow measurement against target B (UNISDR 2017). From the perspective of data availability, feasibility of collection, and measurability, the Expert Working Group recommended the use of a compound indicator based on the number of people injured or ill as a direct result of disasters (B-2) (UNISDR 2017). For details on the computation methodology on death, missing, and affected, please see the Expert Working Group’s report (UNISDR 2017, pp. 8−12).

This report has brought much-needed clarity to national and international organizations that oversee the counting of disaster deaths. Despite this benefit, it is not yet clear if these indicators and definitions have been adopted by the national governments in all low, middle, and high income countries. Nevertheless, according to Maini et al. (2017), the Expert Working Group’s indicators are inspiring, clearly understood, few in numbers, ambitious yet feasible, and, importantly, measurable. The DesInventar Open Source,^1^ which was initiated by the United Nations Office for Disaster Risk Reduction (UNDRR) to monitor the Sendai targets (Mizutori 2020), is a testament to this clarity. Apart from statistical data on the indicators, qualitative research based on people’s opinion about these targets is also in the early stages of development (Ray-Bennett 2018a, 2018b; Green et al. 2019; Ray-Bennett and Shiroshita 2019). Also, there are gaps in the data available on DesInventar, and at times they are patchy. For instance, while reviewing the DesInventar’s Composition of Disasters data, the authors found that within the whole territory of India, only the state of Odisha’s data are available. The Composition of Disasters data is a cumulation of the Spatial Distribution data table and the Temporal Behaviour table data from 1970 to 2014. But DesInventar fails to notify the page reader of this. The Event classifications are also unclear. For instance, “Fire”—is this a forest fire or a domestic fire? It is important that the classifications of the DesInventar Composition of Disasters data are aligned with the indicators of targets A and B (reference Table 1) and with the latest UN Report on Hazard, Definition and Classification Review (UNDRR 2020a).

The enabling conditions of these targets can be understood through existing and emerging theories and frameworks and their complex interlinkages. In the context of this research, targets A and B are the most important of the seven global targets. These targets, especially target A, signal that saving lives during and after disasters is the most essential aspect of disaster risk reduction and disaster risk management practice (Ray-Bennett 2018a, 2018b; Mizutori 2020; Alam and Ray-Bennett 2021). This is also explicit in one of the guiding principles of the Sendai Framework: “Managing the risk of disasters is aimed at protecting persons and their property, health, livelihoods and productive assets, cultural and environmental assets, and the right to development” (UNISDR 2015, paragraph 19(c), p. 13). The authors suggest that this principle is aligned with a human rights-based disaster risk reduction approach, which has its roots in the 1948 Universal Declaration of Human Rights (UN 1948). Currently, all the 192 United Nations member states have ratified this declaration.

Advocates of this Universal Declaration argue that governments and humanitarian actors must integrate a human rights agenda into disaster preparedness, prevention, and response (Brookings-Bern Project 2008; Ferring 2008; Have 2018). This is because four categories of human rights are particularly at stake during disasters, including the right to the protection of life, right to health, right to fulfillment of long term socioeconomic needs, and right to civil and political protection needs (Brookings-Bern Project 2008; Ferring 2008; Have 2018). Of these four categories, the first two are of crucial concern in the immediate aftermath of a disaster because they are prerequisites for other rights. These prerequisites include the protection of security, protection of physical, mental, and moral integrity, protection against violence, including particularly gender-based violence, protection against the negative impacts of natural hazards, protection of rights related to the basic necessities of life, and provision of adequate food, water, shelter and housing, clothing, health services, and sanitation (Brookings-Bern Project 2008; Puspita 2010). All these rights have direct and indirect links to target B’s indicators B-1−B-5 (see Table 1).

Targets A and B are also beginning to be understood through the emerging health in disaster risk reduction framework. Unlike its predecessor, the Hyogo Framework for Action, the Sendai Framework mentions health 38 times (Maini et al. 2017). Health is currently a key theme, and health resilience is promoted throughout the Sendai Framework’s target D (see Paragraphs 18, 30(i), 30(j), 33(c), and 33(o) (Maini et al. 2017). As such, many of the indicators of targets A and B (see Table 1) are health-related—for example, within targets A-1 (number of deaths and missing persons attributed to disasters per 100,000 population) and B-2 (number of injured or ill people attributed to disasters per 100,000 population) (UNISDR 2015; Maini et al. 2017). Within target D the number of health facilities damaged or destroyed, as well as the disruption to health services attributed to disasters, is to be measured (UNISDR 2015; Maini et al. 2017).

Analogous to the above, targets A and B can also be considered through the WHO (2019) Health Emergency and Disaster Risk Management Framework (hereafter, Health-EDRM). The Health-EDRM structure was launched in 2019 to address current and emerging risks to public health, and the need for effective utilization and management of resources at the interface with the Sustainable Development Goals,^2^ the Sendai Framework, and the Paris Agreement.^3^ The vision of the Health EDRM is “the highest possible standard of health and well-being for all people who are at risk of emergencies” (WHO 2019, p. 6). The Health-EDRM is currently touted as an umbrella approach that encompasses emergencies and disaster medicine, disaster risk reduction (DRR), humanitarian response, community health resilience, and health systems resilience (Lo et al. 2017). The Health-EDRM is in its embryonic stage, and its success relies on joint planning and action by the ministries of health and other ministries of national governments, national disaster management agencies, the private sector, communities and community-based organizations, assisted by the international community. At the core of effective Health-EDRM are efforts to strengthen a country’s health system, with a strong emphasis on community participation, as well as action to build resilience and establish the foundation for effective prevention, preparedness, response, and recovery from all types of hazardous events (WHO 2019), and in doing so reduce deaths and morbidities. While this is desired, evidence in the case of the COVID-19 pandemic suggests otherwise. During the first wave of COVID-19, for example, there was no clear evidence that collaboration was taking place between WHO and UNDRR (Djalante et al. 2020).

The interconnection of targets A and B is also strengthened through an efficient disaster risk governance framework. During the first wave of the COVID-19 pandemic, the UNDRR’s Secretary-General, Ms Mami Mizutori, emphasized the importance of saving lives through effective disaster risk governance (Mizutori 2020; Alam and Ray-Bennett 2021). The Hyogo Framework for Action promoted the concept of disaster risk governance through two of its Priorities for Action: “Ensure that disaster risk reduction is a national and a local priority with a strong institutional basis for implementation” (Priority for Action 1), and “Strengthen disaster preparedness for effective response at all levels” (Priority for Action 5) (UNISDR 2005, p. 6). To promote effective disaster risk governance the Hyogo Framework for Action suggested a multisectoral approach and the devolution of disaster management activities, as well as the development of policies, strategies, knowledge, and mutual learning through the Global Platform for Disaster Risk Reduction, the Regional Platforms for Disaster Risk Reduction, and regional cooperation (UNISDR 2005, 2015; Mizutori 2020; Alam and Ray-Bennett 2021).

Disaster risk governance is understood as “the way in which public authorities, civil servants, media, private sector, and civil society at community, national and regional levels cooperate to manage and reduce disaster and climate-related risks” (UNDP 2013, p. 1), which ensures “sufficient levels of capacity and resources are made available to prevent, prepare for, manage, and recover from disasters” (UNDP 2012, p. 1). However, assessments of the performance found that it had a very limited impact on improving governance at the international, national, and local levels, or in reducing social vulnerability to empower particular vulnerable social groups (Schipper et al. 2015). Most importantly, disaster risk governance at the local level has lagged remarkably compared to the national and international levels (Djalante and Lassa 2019; Alam and Ray-Bennett 2021). There is currently no comprehensive data or systematic reporting on the extent to which local governments are implementing disaster risk reduction (Djalante and Lassa 2019), or on their impact in reducing disaster deaths and people affected by disasters (Alam and Ray-Bennett 2021).

Effective disaster risk governance is critical to implementing rights-based disaster risk reduction and Health-EDRM frameworks. This is because these frameworks are fundamentally multisectoral and collaborative, and they promote effective governance to reduce negative health outcomes from all hazards (Lo et al. 2017; WHO 2019). The opinions of the participants for this study concur with these studies and in doing so, the subjective opinions provide a vantage point to analyze the indicators as well as rethink policy and practice relevant to targets A and B. These opinions are presented following the methods section.

Last but not the least, which types of organizations or stakeholders should lead policy and practice relevant to targets A and B. According to the Sendai Framework, while states have the overall responsibility for reducing disaster risk, it is a shared responsibility between government and relevant stakeholders. In particular, non-state stakeholders play an important role as enablers in providing support to states in the implementation of the Sendai Framework at the local, national, regional, and global levels (UNISDR 2015).

The shared responsibility between government and relevant stakeholders to reduce disaster deaths is largely a reflection of the neoliberal values and principles of the globalized world in which we live (Herod 2014). Globalization, coupled with supranational governance arrangements at national and regional levels, means that disaster and development activities are currently delivered partly by governments and partly by nongovernmental organizations (NGOs), civil society organizations (Rooy 2002), multinational companies, and Northern NGOs who prefer to fund Southern NGOs (Bashym 2002).

The Sendai Framework has brought some much-needed clarity to DRR responsibilities, but the framework remains equivocal about who is responsible for reducing disaster deaths and the number of people affected by disasters (Ray-Bennett 2018b; Ray-Bennett and Shiroshita 2019). Empirical studies at the local and national levels that explore accountability and responsibility for reducing disaster deaths are few and far between. Ray-Bennett’s work in the state of Odisha in India (2018a, 2018b) is an exception, which according to the state-level disaster management officials reveals that it is district, block, and village level authorities who are considered to be responsible for reducing disaster deaths. If these actors fail to reduce disaster deaths, there are no legal actions against them because the State Disaster Management Policy (previously known as the Odisha Relief Code) does not specify any legal actions. To achieve targets A and B, more empirical research is needed in this aspect to identify context-specific hindrances, discover enabling factors to overcome them, and support actors who facilitate both.

## Methods, Data Collection, and Data Analysis

The UNDRR’s Global Platforms are used periodically to assess progress, identify gaps, monitor the implementation of the Sendai Framework’s global targets, develop platforms to forge partnerships, share knowledge, and promote the integration of disaster risk management into other relevant sectors (IIASA 2019; UNDRR 2019). Global Platforms are important gatherings of governments, UN agencies, and international and regional organizations and institutions, NGOs, scientific/academic institutions, and private sector entities. In the context of this study, participants who attend these gatherings are referred to here as the global community of DRR. 180 UN member states (among others) attended the Global Platform in 2019 in Geneva (UNDRR 2019). Thus, the Global Platform’s Ignite Stage Forum was a fitting place for this study to collect data to field ideas on targets A and B. Permission was sought from the organizers of the Ignite Stage to collect data during the session. Subsequently, ethics approval was sought for this study from the first author’s base institution. All participants were fully informed of the research’s purpose and conditions. Informed consent was sought from each participant, and they were informed of their right to withdraw from the study at any point of time. The identities of the participants were kept confidential.

Semistructured questionnaires and key informant interviews were conducted to collect participants’ opinions. The pretested questionnaires were distributed to those participants who attended the Ignite Stage session entitled Avoidable Deaths: Translating Sendai’s Goal One into Action and agreed to respond to the questionnaire. Participants were given two options to complete the questionnaire. First, participants could complete the questionnaire manually during the Ignite Stage session. The total duration of the session at the Ignite Stage was 15 minutes. The first half of the session was designed to provide context to the participants, and the latter half of the session was allocated to the participants to complete a paper-based questionnaire. The second option was to complete the questionnaire online using an online survey designed using Qualtrics. For the latter option participants had one month after the session to complete the questionnaire. A total of 55 people completed the questionnaire.

To complement the questionnaire data, semistructured interviews with three key informants were conducted based on convenient and purposive sampling. Prior contact and consent were sought from the informants to interview them at the Global Platform in Geneva. These interviews were conducted after the Ignite Stage session. The key informants included the cofounder of an Indian NGO called Seeds-India,^4^ a Senior Disaster Management Officer from the Office of the Uganda’s Prime Minister, and a Senior Technical Officer from the UNDRR’s Head Office in Geneva. These interviews were recorded in English, and they each lasted between 15 and 20 minutes.

### Data Analysis

The questionnaire had six questions, of which three were closed and three were open questions. The closed questions were in the form of multiple-choice questions with a few options from which to choose depending on the question. For the open-ended questions, the participants had the opportunity to write a sentence or two on their opinions on the subject matter. Section one of the questionnaire collected background information about the participants, while section two gathered perspectives and opinions on whether it is possible to achieve global targets A and B and reduce avoidable deaths from disaster events. Analysis of the questionnaire data was carried out in three steps using Microsoft Excel and the Statistical Package for the Social Sciences (SPSS) version 13. Microsoft Excel was mainly used for the initial data analysis and for creation of the corresponding bar graphs. SPSS was used to cross check the figures through the use of frequency tables. Comparisons were made between gender, years of experience, education level, type of organization employing the informant, and country development status.

The first step in the analysis was to study the differences in baseline characteristics by noting the frequency of the answers given, inclusive of the subjective data. This was also further examined by noting the mode or the most frequently occurring answer or variable. The mode is a measure of central tendency, which is important for the type of categorical data that this study utilizes.

In the second step, group level analysis was undertaken using comparison demographics to show their impact on the opinions of the participants. This was done using multivariate analysis, namely the correlation calculation, to see how well any combination of any two variables (question 1 against question 2) relate. A positive correlation would mean a positive relationship, 1 being the highest. The opposite would correspond with negative correlation, which means that the two variables move in the opposite direction, -1 being the most negatively correlated. However, due to the nature of the study, and the sample size (55), a regression analysis was not possible since this technique requires a larger sample size to achieve a statistically significant p-value. The correlation was calculated using the Excel formula CORREL, which calculates a simple correlation coefficient.

In the third step, an analysis of the overall trends or relationships was completed. This was done using comparative thematic analysis (Boyatzis 1998). This was especially helpful in the assessment of the open-ended questions by highlighting key words that reoccurred for the answers given. Each variable was analyzed using a univariate approach. This was then combined with a multivariate approach. That is, a cross analysis of the answers given was done by highlighting any notable trend that was often supported with popular literature. However, no real conclusive relationships were found while conducting this approach based on the data obtained from the sample. The data from three key informant interviews are provided for side-by-side analysis of the questionnaire data (Creswell 2014).

### Limitations of the Data

The sample size for this study is small. This is partly because the study was designed exclusively for the participants who attended the specific session Avoidable Deaths: Translating Sendai’s Goal One into Action at the Ignite Stage, and partly because this was an exploratory study designed to bring a fresh departure to the traditional presentation raising awareness of the Sendai Framework’s first two targets. Using a nontraditional presentation method was one of the criteria by the UNDRR’s Review Team for securing a 15-minute slot to speak at the Ignite Stage. To the best of the authors’ knowledge, participants attending the Global Platform for DRR register for the whole event rather than for specific sessions. Therefore, it is difficult to mention how many participants registered for the Global Platform in 2019, and what proportion of them attended the Ignite Stage for this specific session. Parallel sessions were ongoing at the Global Platform when the first author spoke at the Ignite Stage and conducted the quick survey for this study. The Ignite Stage event was conducted in an open space rather than in a hall or a room, with 30−40 chairs for audience members. Although the first author had two assistants to assist with the distribution and collection of the questionnaire during the session, the session was too short and intense to develop a precise headcount.

Although the data are not a full representation of the global community of DRR opinions concerning targets A and B, the data meet the need for this small-scale, qualitative study. Wherever possible, secondary data have been used to triangulate findings in order to increase the validity of the data. However, the purpose of this study was to gain insight into informed opinion and to improve understanding of the Sendai targets by exploring obstacles that obstruct meeting 2030 targets, and identify those variables that may promote attainment of Sendai goals at the national and local levels. Although in-depth interviews over a month or two would have generated rich data in a different set-up, the data collected were detailed enough to solicit ideas to reimagine the interconnections of targets A and B with rights-based, health and disaster risk governance frameworks. The data set also was sufficiently robust to recommend policy and practice improvement for UNDRR and UNDP, organizations that are mandated to support national governments in the reduction of disaster deaths and the decrease in number of people affected by disasters.

## Results

This section provides the results of the questionnaire and three key informant interviews specific to our two research questions.

### Can Targets A and B Be Achieved and What Key Factors Lead You to Your Response?

Of the 55 participants in the questionnaire survey, 16 were female (29%) and 39 were males (71%). These participants represented 24 different countries from both the high income and low-middle income categorization of countries. Fifteen participants were from academia, 12 came from governmental organizations, 10 represented nongovernmental organizations operating at various levels and community-based organizations (CBOs), six were from the private sector, four were from the United Nations, one was from a research institute, one was a consultant, and six participants identified with some combination of the different fields listed above. With respect to work experience in a disaster-related field, 22 participants had 6−15 years of experience, 17 had 0−5 years of experience, nine had more than 15 years of experience, three were students, and two participants were not from the disaster-related field. Twenty-four participants had an advanced degree (for example, MSc), 18 had a doctorate (for example, PhD), seven had first degrees (for example, BA, BSc), and five participants had other qualifications (for example, diploma, certificate). One participant did not answer. This indicates that more than 76% of the participants were postgraduates, and 58% had more than six years of experience in a disaster-related field.

Most (83.3%) of the participants, as well as the three key informants, said “yes” that target A can be achieved by 2030, and the key factors for this opinion (in descending order) are: disaster risk reduction efforts, early warning systems, awareness, finance and investment, climate change, policy, technology, coordination, *collaboration, human security, governance communication,*^5^* funding, poverty, inequality, population control, safety nets, social resilience, capacity building, and mitigation strategies.*^6^ Less than a quarter (16.7%) of the participants said “no,” target A cannot be achieved, and the key factors for this are listed in Box 1A.

**Table:** 

*Box 1: Key factors for not achieving target A*
Respondent #3: Minimal investment on human security.
Respondent #6: Response and coordination needed to achieve the target may require a longer timeframe.
Respondent #7: Rising temperatures, erratic weather patterns due to climate change, leading to increased uncertainty and death. I do not see the spread of technology to reduce deaths extending to the poor and vulnerable.
Respondent #8: There is not enough information sharing and collaboration between practitioners.
Respondent #15: Governments haven’t taken actions to reduce the risk of disaster, especially in developing countries where the lack of resources and low investment in disaster risk reduction is a huge obstacle.
Respondent #16: Climate change effects on geohazards are still poorly understood. Urban planning that takes into account natural hazards is far behind in developing nations.
Respondent #53: Conflict and violence are often under-thought about in terms of a long-term vulnerability contributing to mortality in different ways and ever.
Respondent #55: Not enough action by the government since Sendai Framework is voluntary.

Just over 80% of the participants and the three key informants said “yes” when asked whether B can be achieved, and the key factors identified for this outcome (in descending order) are: awareness, early warning, *investment, targeted investment, targeted infrastructure,*^7^* information, planning and funding,*^8^* climate change and uncertainty, technology development, empowerment, energy security, engagement,*^9^* budget allocation, desertification, health security, human security, sensitization and involvement.*^10^ The remaining 19% of the participants said “no,” concluding that target B cannot be achieved, and citing the key factors for this conclusion that are presented in Box 2.

**Table:** 

*Box 2: Key factors for not achieving target B*
Respondent #11: Poor information, lack of campaigns and low budget allocation in development economies.
Respondent #15: Climate change is increasing the number and intensity of disasters, plus low investment in resilient and social infrastructure.
Respondent #20: 1. […]. Even if improved early warning mechanisms alert governments to evacuate and save millions of lives, the number of people affected by the phenomenon would still be pretty high. For instance, during the recent Cyclone Fani, a number of casualties was minimal but the number of affected and evacuated people was very high. 2. Increasing desertification due to global warming will lead to more protracted droughts thereby affecting yields, food prices and food security of millions of people. 3. Climate change uncertainty […] makes planners and policymakers ill-equipped to devise effective countermeasures to protect at-risk communities, thereby increasing the number of affected people.
Respondent #55: More hazards, denser population, more settlement, lead to more affected people.
Respondent #25: It takes more than one person to implement changes. In order to achieve such goals, many countries need to accept the reality and want change. Unfortunately, besides being corrupted by power, money and greed, government stakeholders lack the basic understanding and show care to change the effects.

### Results of Target A versus Target B

Circa 75% of the participants who said that target A could be achieved also said that target B could be achieved. Only 12% of the participants said “no” for both targets A and B. The correlation recorded a positive affirmation of both targets A and B and was approximately 0.55, which shows a reasonably positive correlation. This is expected since the variable, development status of the participant’s country, was constant and both targets A and B were similar, dealing with mortality rate or people affected by 2030, respectively. Development status is simply a general classification of the participant’s country on whether the country is a low, lower-middle, upper-middle, or high-income country as per the World Bank’s classifications (Hamadeh et al. 2021). Therefore, it stands to reason that the opinions would be alike and correlate since the variables or targets themselves under comparison were not particularly dissimilar.

Optimism about achieving the targets varied slightly based on how experienced the participants were in a disaster-related field, with 83% of all participants thinking that target A was achievable, while 17% disagreed. All of the participants who had 15+ years of experience believed that it is possible to achieve target A. In addition to this, 100% of participants who indicated that disaster risk management was not their primary field felt that target A was not achievable. The correlation recorded was approximately 0.2 or a slightly positive correlation between years of experience and support for target A being achievable. In analyzing the income status of the participant’s country, 85% of the participants from the low-middle income bracket believed that target A was achievable, while a slightly smaller proportion (79%) from the high income bracket thought that it was achievable. The correlation recorded was approximately 0.08, which is negligible and shows very little to no correlation.

On achieving target B, overall, 80% of the participants believed that a successful result is achievable. Approximately 20% of the participants thought that target B could not be achieved. This optimistic number is slightly less than the 83% that was recorded for target A. Also, the mode for the dataset “key factors” is awareness, so it is reasonable to state that most participants believed that awareness is a key factor in successfully achieving target B goals.

In analyzing the effect of the income status of the Participants, exactly 75% of the participants from high income countries believed that target B was achievable, while 82% from low-middle income countries thought that it was achievable. Overall, there were 41 participants from low-middle income countries compared to 14 from high income countries.

With the participants categorized by years of experience in a disaster-related field, 80% of all participants thought that target A was achievable, while 20% disagreed. This difference (83% versus 17% above) occurred because some participants did not answer the experience query. There were participants who indicated target A was unachievable in each category of experience, but this opinion was most prevalent in the mid-career cohort (6−15 years’ experience). The correlation recorded was approximately 0.195, indicating a slightly positive correlation between years of experience and support for target B being achievable.

Comparing the responses to the mode of the “years of experience” dataset, it can be concluded that the correlation indicates that most participants who said “yes” are mid-career in the disaster management field. It is further possible to infer that seasoned persons in the height of their respective fields may share increased optimism in the achievement of these goals.

### What Is the most Effective Way to Bring about a Global Reduction in Disaster Deaths?

**Fig. 1:**
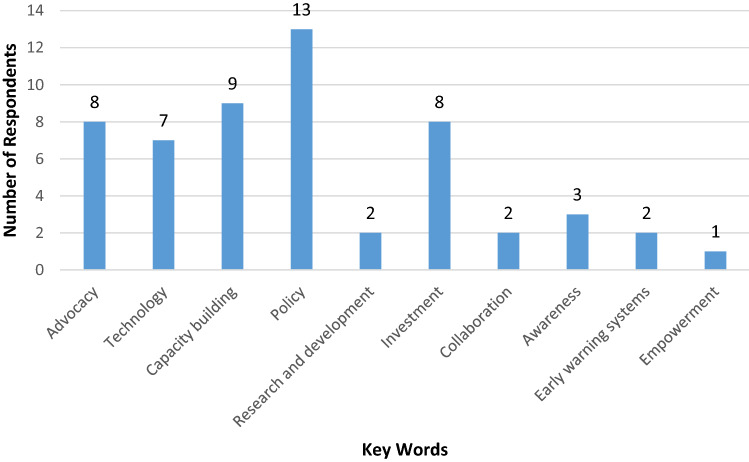
Effective ways to bring about a global reduction in disaster deaths

Exactly 55 responses were received. The responses for effective ways to bring about a global reduction in disaster deaths (in descending order) are: policy, capacity building, investment, advocacy, and technology (Fig. 1).

The mode of this dataset was compared with the mode of the dataset of the current occupation of the participants. Based on the mode comparison of these two datasets, it can be deduced that there is a correlation between the participants’ occupations, and perceived ways to achieve a global reduction in deaths. However, given the relatively small sample size, there are limitations to these conclusions and a more tailored study would be required to confirm these views. From the answers collected, a significant portion of the professor level academic sector believed that policy is the most effective way to bring about a global reduction in avoidable deaths from disaster events. Policy in this sense relates to disaster-related responses, building codes, and other specific disaster mitigation governance tools or procedures. It must also be noted that capacity building, advocacy, investment, and technology are also noted as important to a global reduction of deaths.

Comparing the modes for the participants’ organizations (government and NGO/CBO), a correlation can be deduced between the responses for the most effective way to bring about a global reduction. Participants in our survey believed that policy is the most effective way, and this is likely given the nature of the work of the government and nongovernmental sectors in which participants engage.

### Which Type of Organizations Should Take the Lead in a Country’s Effort to Reduce Disaster Deaths?

**Fig. 2:**
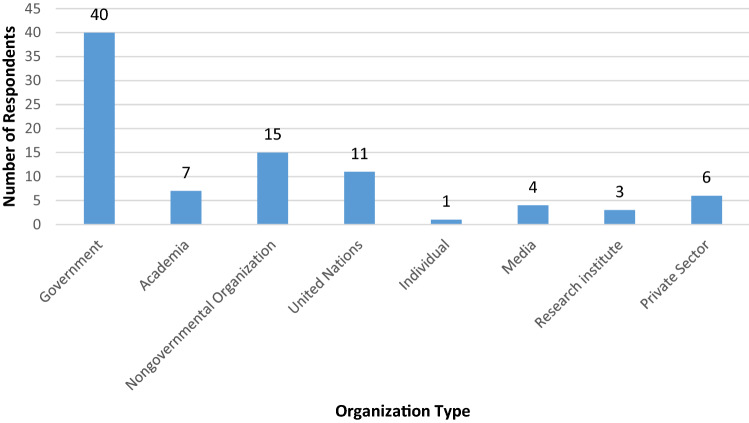
Organizations that can lead national efforts to reduce disaster deaths

Forty participants thought that the national government should take the lead in a country’s effort to reduce disaster deaths (46%), followed by 15 participants for NGOs (17%), the UN with 11 participants (13%), academia seven participants (8%), private sector six participants (7%), the media four participants (5%), research institutes three participants (3%), and one individual (1%) (Fig. 2). The participants for this question in some cases selected more than one answer, thereby inducing a total number of responses over the 55 participants in the survey. In all 87 responses were recorded.

Comparing the modes of the lead organizations in reducing disaster deaths and the participants’ organizations, it can be deduced that there is a correlation between the two. As most participants are from academia and government sectors, it is not surprising that the government is listed as the lead organization in leading a country’s effort to reduce disaster deaths. This can be attributed to personal biases originating from the population’s professional background, as well as a desire to support the Sendai Framework’s effort to bring clarity to DRR.

## Discussion

This section discusses the findings in relation to existing literature wherever we could.

### Conditions that May Facilitate and Hinder the Achievements of Targets A and B

Participants identified 20 key factors that can facilitate the achievement of target A. Of these 20 factors, the top 10 key factors are: DRR, awareness, early warning systems, investment/finance, climate change, policy, technology, coordination, communication, and governance. All these key factors fall within the remit of the Sendai Framework’s disaster risk reduction framework. The importance of the top 10 key factors is also consistent with several previous studies. For instance, the early warning early action program (EWEA) saved lives when the Barge Dam in Ghana spilled (Braman et al. 2013); space-based early warning systems coupled with effective evacuation measures reduced disaster deaths in the Indian state of Odisha from > 10,000 during the super cyclone of 1999 to 86 fatalities during cyclone Phailin in 2013, and to 41 storm-related deaths during cyclone Fani six years later (Ray-Bennett 2018a, 2018b). In Bangladesh, effective risk communication through an early warning system led to reduction in disaster deaths from circa 300,000 in 1970 to 3,000 deaths as a result of cyclone Bhola in 2007 to 26 deaths during the super cyclone Amphan in 2020 (Fakhruddin et al. 2020).

Finance and investment, policy development, and improved technology are equally important to reduce disaster deaths (Ray-Bennett 2018b; Mizutori 2020). But action in these areas is currently lagging. According to the World Bank (2022), without significant investment into making cities resilient, areas where more than two-thirds of the world’s population will live by 2050, worldwide disasters may cost cities USD 314 billion each year. Investing in more resilient infrastructure can provide a net benefit in low and middle economic status countries by improving the quality and resilience of essential services such as transport or water and electricity supply, which can contribute to more resilient and prosperous societies (World Bank 2022).

Unfortunately, investment has not been matched with the increasing number of disasters, disaster losses, and rapid urbanization, especially in low to middle income countries. This is echoed by Mami Mizutori (2020, p. 150), UNDRR’s Special Representative of the Secretary-General for DRR:

The biggest challenge […] we face is the lack of greater and more direct funding to achieve the Sendai Framework and to reduce disaster risk. The SFM (Sendai Framework Monitor system) data showed that at end-September 2019, 15 recipient countries had reported receiving a mere USD 1.5 billion in bilateral and multilateral aid for disaster risk reduction.

The participants who believed that target A cannot be achieved also highlighted “minimal investment” (participant #3), and lack of government “actions to reduce the risk of disaster especially in low-middle income countries […] and low investment in disaster risk reduction […]” (participant #15, participant #55) as the key hindrance factors.

The participants identified 18 key factors that can facilitate achievement of target B. The top 10 are: awareness, early warning, investment, infrastructure, targeted investment, planning and funding, climate change uncertainty, technology development, empowerment, and energy security. The participants who did not believe that target B can be achieved identified several key hindrance factors for this unfortunate future. These were a “lack of campaigns and low budget in development economies” (participant #11), “climate change […] low investment in resilient and social infrastructure” (participant #15), “[…] climate change uncertainty […which] makes planners and policymakers ill-equipped to devise effective countermeasures to protect at-risk communities, thereby increasing the number of affected people” (participant #20). It was noted that some of these responses overlap with the responses for target A (for example, investment, early warning) that reflect people’s lived experiences.

Despite an increase in the number of hazards (approximately 90 annually in the 1970s to approximately 450 annually in the 2000s), the number of disaster deaths has decreased (IFRC 2013; UNISDR 2015). At the same time the number of people affected by disasters has increased (target B). This is emphasized by participant #20 (see Box 2), and is echoed by the UNISDR report (2018, p. 3), which states that “the direct economic loss incurred by the disaster-hit countries between 1998 and 2017 is valued at US$ 2,908 billion […] with disasters pushing 26 million people into poverty every year.” Although we do not fully understand the depth and breadth of disaster impacts on households, it is certain that they are multifaceted and relevant to humanitarian concerns. The participants emphasized a wide range of key factors that can facilitate achieving the Sendai Framework’s targets, including early warning systems, infrastructure, investment, energy security, human security, and addressing climate uncertainty. These key factors are important to realize the targets, but at the same time they cut across sectors, policies, and practices, which is consistent with the Hyogo Framework/Sendai Framework’s multisectoral approach to reduce the number of disaster deaths and people affected by disasters (UNISDR 2005, 2015).

Despite this, national and local governments may find it difficult to implement a multisectoral approach to targets A and B. For example, the disaster management structures of India (Ray-Bennett 2018a, 2018b), Bangladesh (Alam and Ray-Bennett 2021), Japan (Ray-Bennett and Shiroshita 2019), Uganda and Kenya (Corsel et al. 2017) are not agile enough to go beyond the disaster and development sectors to include energy, health, housing, critical infrastructure, and other sectors, for instance. To realize target B, it is pivotal that these sectors work together in everyday life through standard operating procedures to reduce the number of people affected by disasters directly and indirectly. If the impact of a disaster on the affected people is not reduced, it is likely that this will lead to indirect or secondary disaster deaths. For instance, in Japan there were 50 direct deaths and 212 indirect deaths in the Kumamoto Earthquake in 2016, and 13 direct deaths and 224 indirect deaths in flooding in 2018 (Japan Times 2019). Therefore, it is pivotal to consider the number of indirect deaths in order to improve our understanding of how best to achieve target B.

### Most Effective Ways to Reduce Disaster Deaths

The participants identified 10 effective ways to achieve a global reduction in disaster deaths (see Fig. 1). The top five ways are: policy, capacity building, *investment* and *advocacy*,^11^ and technology. These are consistent with the wide spectrum of the Sendai Framework’s disaster risk reduction framework and disaster risk governance. The year 2020 was the year to achieve the Sendai Framework’s global target E, which emphasizes governance by “Substantially increase[ing] the number of countries with national and local disaster risk reduction strategies” (UNISDR 2015; UNDRR 2020b). According to the UNDRR’s Status Report as of August 2020, 93 countries achieved target E and 100 countries (52%) did not. As such, “to accelerate efforts to reach Target E by the end of 2020 remained an urgent priority” for the UNDRR (UNDRR 2020b, p. 7).

The participants for this study also emphasized the importance of building up the capacity of disaster responders. In this regard, the UNDRR’s Global Education and Training Institute (GETI) is providing support to achieve target E through training and webinars. As of October 2020, they have trained 8,402 persons. More is needed to facilitate the achievement of targets A and B at the national and local levels. It is evident that targets A and B are interconnected with the global target E (disaster risk governance and capacity building) (UNISDR 2015; Alam and Ray-Bennett 2021). Achieving progress on target E simultaneously improves targets A and B. As such, it is imperative that these three targets are understood and supported in tandem by the UNDRR and national governments.

### Organization that Should Lead in Reducing Disaster Deaths

According to the participants, national governments should lead efforts to reduce disaster deaths, followed by the NGOs and the UN. These opinions align with the Sendai Framework’s principle of shared responsibility between government and relevant stakeholders.

Despite this, the opinions of the participants indicate that it has to be the national governments who take on this responsibility. It is likely in case of a national-level disaster, however, that transnational coordination and cooperation may be necessary, but this was not emphasized by the participants. Furthermore, other stakeholders such as churches, defence forces, emergency services, civil society, and individuals who play a key role in reducing disaster deaths were not mentioned by the participants. In fact, “Individual” or regular citizens was ranked the lowest (1%) in reducing disaster deaths (Fig. 2). This may be because these stakeholders are underrepresented at the Global Platform for DRR and the participants who attended the specific session at the Ignite Stage in 2019 reflected that fact. This has implications for the Ignite Stage and the Global Platform to be more inclusive and attract a more diverse gathering.

## Conclusion

A few recommendations are outlined to reconfigure policy, practice, and research for targets A and B. Increasing the number of disaster risk reduction strategies and policies is likely to increase investment in science (social and physical), awareness, and empowerment of disaster responders and at-risk communities. However, target E was not achieved in 2020 by 100 UN member states (UNISDR 2017). Therefore, it is paramount that the UNDRR, UNDP, and regional organizations provide support to the remaining member states to achieve target E and, in doing so, assist targets A and B to be realized.

It is also evident that targets A and B warrant a multisectoral approach. Based on the participants’ responses, six key sectors are apparent: health, climate change, poverty/human security, energy security, housing, and critical infrastructure. National and local governments with limited resources and know-how may find it difficult to implement multisectoral approaches (Djalante and Lassa 2019; Alam and Ray-Bennett 2021). To overcome these limitations, the relevant stakeholders, including UNDRR, UNDP, regional organizations, GETI (Global Education and Training Institute), and national governments can promote collaboration and coordination across the six sectors to develop an agenda for a multisectoral approach and an implementation plan for targets A and B.

As the number of disaster deaths continues to decrease due to effective disaster risk management practices, local and national-level disaster risk governance, and early warning systems (Ray-Bennett 2018a; Fakhruddin et al. 2020; Mizutori 2020), it is critical to focus on target B (particularly indicators B-1 to B-5) because the number of people affected by disasters has gone up by 151% in the period between 1998 and 2017 (UNISDR 2018). If target B is not sufficiently emphasized by UNDRR, UNDP, and national and regional organizations, the consequence is likely to be an increase in the numbers of indirect deaths, and thus poverty and vulnerability will ratchet upward in low and middle income countries. Currently mechanisms to document indirect deaths are lacking. As research and methods to capture indirect deaths through passive surveillance and a combination of epidemiological and social science methods (for example, verbal autopsy, social autopsy, and in-depth interviews) are required. Credible death tolls (both direct and indirect) are important for community recovery after disasters, can enhance political accountability, and are also vital to guide better-informed disaster, livelihood, and public health policies (Guha-Sapir and Checchi 2018).

It is equally important for UNDRR, UNDP, and regional organizations to highlight the importance of national governments in realizing targets A and B. The domain of disaster risk reduction is crowded by many actors and organizations. A crowded domain of multiple stakeholders can displace the focus from the national governments to other actors. This is neither conducive for rights-based disaster risk reduction to promote accountability and justice for the members of the deceased families, nor conducive to the WHO Health-EDRM’s vision. Thus UNDRR, UNDP, WHO, and regional organizations must reemphasize the importance of national governments’ responsibilities, including heads of state, in achieving targets A and B. This can be done through the UN’s global/national platforms and bilateral agreements (among other things). The emphasis on national governments and political leaders’ responsibilities will lead to investment in developing their capacities through knowledge sharing and continuous professional development training on methodology, accountability, multisectoral approaches, social justice, and sustainable development. The authors are, however, conscious of the fact that this recommendation may not be applicable to the all leaders of nation states, especially those who are currently embroiled in conflict or war, as well as those who are not UN member states.

## Footnotes

1 https://www.desinventar.net/

2 https://sdgs.un.org/goals

3 https://unfccc.int/process-and-meetings/the-paris-agreement/the-paris-agreement

4 https://www.seedsindia.org/

5 The Italicd variables received same percentage.

6 The Italicd variables received same percentage.

7 The Italicd variables received same percentage.

8 The Italicd variables received same percentage.

9 The Italicd variables received same percentage

10 The Italicd variables received same percentage.

11 Investment and advocacy received same percentage.
